# HBX-6, Standardized *Cornus officinalis* and *Psoralea corylifolia* L. Extracts, Suppresses Benign Prostate Hyperplasia by Attenuating E2F1 Activation

**DOI:** 10.3390/molecules24091719

**Published:** 2019-05-02

**Authors:** Bo-Ram Jin, Hyo-Jung Kim, Jong-Hwan Seo, Myoung-Seok Kim, Kwang-Ho Lee, Il-Joo Yoon, Hyo-Jin An

**Affiliations:** 1 Department of Pharmacology, College of Korean Medicine, Sangji University, 83 Sangjidae-gil, Wonju-si, Gangwon-do 26339, Korea; wlsqh92@gmail.com (B.-R.J.); hyojung_95@naver.com (H.-J.K.); sjhjej@naver.com (J.-H.S.); 2 Central Research Institue of Hawon Pharmaceutical, Jangheung 59338, Korea; mskim1210@naver.com (M.-S.K.); gusins0930@naver.com (K.-H.L.); polorman@naver.com (I.-J.Y.)

**Keywords:** BPH, RWPE-1, WPMY-1, E2F1, *Cornus officinalis* Sieb. et Zucc., *Psoralea corylifolia* L., HBX-6

## Abstract

Background: The aim of this study was to simplify and identify the contents of the herbal formula, HBX-5. This study was carried out to evaluate the therapeutic effects of HBX-6 in a mouse model of benign prostatic hyperplasia (BPH). Based on in vitro, we selected a candidate, reconstituted an experimental agent and investigated the effects on testosterone-induced BPH rats. Cell viability was determined by MTT assay in RWPE-1 and WPMY-1 cells. The expression of androgen receptor (AR) was measured in dihydrotestosterone-stimulated RWPE-1 and WPMY-1 cells. BPH was induced in mice by a subcutaneous injection of testosterone propionate for four weeks. Animals were divided into six groups: Group 1, control mice; Group 2, mice with BPH; Group 3, mice with BPH treated with finasteride; Group 4, mice with BPH treated with 200 mg/kg HBX-5; Group 5, mice with BPH treated with 100 mg/kg HBX-6; and Group 6, mice with BPH treated with 200 mg/kg HBX-6. Changes in prostate weight were measured after treatments, and the thickness of the epithelium was evaluated. The expression levels of proteins associated with prostatic cell proliferation and cell cycle-related proteins were determined. Based on previous reports and in vitro results, we selected *Cornus officinalis* and *Psoralea corylifolia* among HBX-5 components and reconstituted the experimental agent, and named it HBX-6. The result represented a new herbal formula, HBX-6 that suppressed the pathological alterations in BPH and showed a marked reduction in proliferation-related protein expression compared to mice with BPH. Our results indicate that HBX-6 has a better therapeutic effect in the BPH murine model than those of HBX-5 and finasteride, suggesting the role of HBX-6 as a new BPH remedial agent.

## 1. Introduction

Benign prostatic hyperplasia (BPH) is one of the most frequently reported male health disorders, and has a considerable impact on men older than 50 years worldwide. The cumulative prevalence of BPH has been shown to range from 50% in men aged 41–50 years and to increase by 10% per decade and reach 80% in men older than 80 years. Most men older than 80 years are likely to experience the pathological symptoms of prostatic hyperplasia [1]. BPH is defined as a nonmalignant overgrowth prostate condition, which is implicated in lower urinary tract symptoms (LUTS) and bladder outlet obstruction (BOO) [2,3]. While there has been some agreement on the etiology of BPH, many researchers have reported that several risk factors, such as ageing, excessive dihydrotestosterone (DHT) levels, and the alteration of hormones may be involved in the development of the disease [4,5]. One major issue in BPH research is concerned with the interaction between hormonal disturbance and cellular proliferation [6]. Based on histological diagnosis, BPH has been characterized by the unregulated proliferation of connective tissue, smooth muscle, and glandular epithelial cells [7]. During BPH development and progression, cellular proliferation leads to prostate enlargement and the augmentation of stromal smooth muscle tone [8].

BPH has best been treated by two major categories of drug: α1-adrenergic receptors blockers and 5α reductase inhibitors. Alpha1 blockers bind and block the cognate receptors and relax the prostatic smooth muscle, relieving BOO [6]. Five alpha reductase inhibitors, also called DHT blockers, have primarily been used in the treatment of BPH. These agents prevent the conversion of testosterone to DHT, leading to prostate volume shrinkage and mitigation of urinary tract symptoms. While these agents are effective at symptomatic improvement, a significant limitation of these drugs is their adverse effects, such as reproductive dysfunction, gynecomastia, and subsequent progression to prostate cancer [9]. Hence, there is a definite need to develop substitutes for these drugs with reduced side-effects. As part of these efforts, herbal medicine-based drug development has been proposed.

HBX-5 is a standardized herbal medicine-based formula suggested for the treatment of BPH and is formulated from nine medicinal herbs. Our previous findings showed the antiproliferative effects of HBX-5 in a testosterone-treated rat model and suggested that HBX-5 could be further explored as a potential herbal medicine for the treatment of BPH [10]. Although our previous investigation indicated the therapeutic potential of HBX-5 in BPH development, medicine preparation process was limited by the complexity of HBX-5 composition, which suggested the need to simplify the contents of HBX-5.

Here, we established a DHT-stimulated prostate cell model to evaluate the inhibitory effect of individual component herbs of HBX-5 on androgen receptor (AR) expression. Based on in vitro results, we selected *Cornus officinalis* Sieb. et Zucc. and *Psoralea corylifolia* L., and reconstituted the new herbal formula, HBX-6. After the formulation of HBX-6, we identified its representative chromatograms. Based on the HPLC analysis and previous studies, we evaluated the antiproliferative effect of HBX-6 in testosterone-treated mice. Oral administration of HBX-6 suppressed prostate enlargement and pathological changes induced by testosterone injection through inhibition of proliferation-related protein expression. This molecular mechanism is associated with the inhibition of the E2F1–Rb pathway and a reduction in cyclin D1 expression. Overall, our study presents the possibility of treatment of BPH by the antiproliferative effect of new combined formula, HBX-6.

## 2. Materials and Methods

### 2.1. Chemicals and Reagents

Testosterone propionate (TP) was purchased from Wako Pure Chemicals (Tokyo, Japan). Finasteride was supplied from Merck & Co., Inc. (Kenilworth, NJ, USA). Antibodies against androgen receptor (AR, sc-816), proliferating cell nuclear antigen (PCNA, sc-56), E2F1 (sc-193), Rb (sc-377528), cyclin D1 (sc-753), and β-actin (sc-81178) were obtained from Santa Cruz Biotechnology, Inc. (TX, USA). Antibody against prostate-specific antigen (PSA, Catalog number, PB9259) was purchased from Boster Biological Technology (CA, USA). Loganin (≥97.0%), psoralen (≥99.0%), and isopsoralen (≥95.0%) were procured from Sigma-Aldrich (Seoul, Korea). Morroniside (≥97.0%) was purchased from ChemFaces (Wuhan, Hubei, China). HPLC-grade acetonitrile and water were procured from JT&Baker (Seoul, Korea).

### 2.2. Cell Culture and Sample Treatment

The normal human prostatic epithelial cell line, RWPE-1, and normal human prostatic stromal cell WPMY-1 were acquired from the American Type Culture Collection (Manassas, VA, USA). RWPE-1 cells were cultured in Keratinocyte Serum-Free Medium supplemented with 0.05 mg/mL bovine pituitary extract, 5 ng/mL human recombinant epidermal growth factor, and an antibiotic-antimycotic cocktail (Gibco, Grand Island, NY, USA). WPMY-1 cells were cultured in Dulbecco’s Modified Eagle’s Medium, supplemented with 1% penicillin/streptomycin and 10% FBS (Gibco). After 24 h of incubation, the cells were serum starved prior to some of the experiments, as indicated. Then, the cells were treated with 10 nM DHT for 24–72 h, with or without various concentrations of components from HBX-5 (0.25–1000 μg/mL).

### 2.3. Cell Viability Assays

Cells were treated with the herbal components (0.25–1000 μg/mL) and incubated overnight followed by the addition of MTT solution (5 mg/mL) for 2 h. After aspirating the supernatant, the formazan product was dissolved in DMSO, and extent of cytotoxicity was measured at 570 nm using a BioTek™ Epoch microplate spectrophotometer (Winooski, VT, USA).

### 2.4. Preparation of the HBX-6

*Cornus officinalis* Sieb. et Zucc. and *Psoralea corylifolia* L. were obtained from Hwapyung D&F Co., Ltd. (Seoul, Korea). HBX-6 composition was as follows (values indicate proportions of each ingredient, expressed in parts for 1000 g): *Cornus officinalis* Sieb. et Zucc. (650 g) and *Psoralea corylifolia* L. (350 g). These two crude drugs were decocted gently in 10 times volume of 30% ethanol for 3 h and filtered, and the powder obtained was spray-dried to yield an extract that was approximately 8.33% of the original preparation by weight.

### 2.5. Chromatography Conditions

Analyses were performed using a C18 column Luna^®^ (Phenomenex, Torrance, CA, USA) (250 × 4.6 I.D. 5 μm particle size) protected by a disposable security guard precolumn (3.0 × 4.0 mm), and was maintained at 35 °C, using a thermostatically controlled column heater. The mobile phase was filtered through a polyvinylidene difluoride (PVDF) membrane (0.2 mm, PALL^®^ Corporation, Port Washington, NY, USA) and degassed using an ultrasonic bath. Samples and analytical standard solutions were previously filtered through a 0.2 mm Polytetrafluoroethylene membrane (Sartorius^®^, Göttingen, Germany). The mobile phase consisted of water containing 10 mM ammonium acetate (pH 6.75, A) and acetonitrile (B). A gradient elution was programmed as follows: 0–5 min, 5.0%–5.0% (*v*/*v*) B; 5–55 min, 5%–80% B; 55–62 min, 80%–90% B; 62–64 min, 90%–5% B; 64–69 min, maintaining 10% B at a flow rate of 1.0 mL/min. Morroniside, loganin, psoralen, and isopsoralen were detected at 240 nm. Injection volume was 10 μL.

### 2.6. Calibration Curves, Limits of Detection, and Limits of Quantification

A 70% methanol stock solution containing the 6 reference components was prepared and diluted to appropriate concentrations for the construction of calibration curves. Six concentrations of the mixed standard solution were injected in triplicates, and their regression values were calculated by the equation Y = AX + B (Table 1). The dilute solution was further diluted to a series of concentrations with 70% methanol for the gain of the limits of detection (LOD) and limits of quantification (LOQ). The LOD and LOQ under the indicated chromatographic conditions were determined at a signal-to-noise (S/N) ratio of 3 and 10, respectively.

### 2.7. Animal Treatments

A total of 60 male ICR mice (25 ± 2 g) were acquired from Daehan BioLink (Eumsung, Korea). All the experimental protocols were performed according to the IACUC Animal approval protocol from Sangji University prior to the initiation of any experimental study (#2017-02). BPH was induced in the mice by intramuscular injections of testosterone propionate for 30 days, as reported previously [11]. Briefly, mice were divided into six groups: control; BPH-induced mice (BPH); BPH induced mice treated with finasteride (5 mg/kg/day), p.o.; BPH-induced mice with HBX-5 200 mg/kg/day, p.o. (HBX-5 200 mg/kg); BPH-induced mice with HBX-6 100/200 mg/kg/day, p.o. (HBX-6 100 mg/kg and HBX-6 200 mg/kg, respectively). After 24 h of the final injection, body weights of all animals were measured, and the animals were sacrificed. The entire prostate tissues were resected and weighed for the PW/BW ratio which was computed as described below.

PW/BW ratio = (prostate weight in each mice of the experimental group/body weight in each mice of the experimental group) × 1000

### 2.8. Histological Analysis

The prostate tissues in each group were fixed with 4% formalin and embedded in paraffin, and the tissues were cut and stained with hematoxylin and eosin (H & E) for histological examination. Images were acquired using a Leica microscope (Leica DFC 295, Wetzlar, Germany).

### 2.9. Western Blot Analysis

The prostate tissues were lysed, and proteins were extracted using the lysis buffer. After extraction, proteins from each experimental group were separated on 8–12% polyacrylamide gels and were transferred on to PVDF membranes. After blocking, the membranes were incubated in primary antibodies described above. Followed by primary antibody incubation, the antibody was removed by washing the membranes thrice with TBST and the membranes were then incubated for 2 h with horseradish peroxidase-conjugated secondary antibody (1:2500) at 25 °C. After incubation, membranes were washed thrice with TBST, and the immunoreactive bands were detected using ECL solution (Ab signal, Seoul, Republic of Korea) and were captured on an X-ray film (Agfa, Belgium).

### 2.10. Statistical Analyses

The experimental data presented are represented as the mean ± standard deviation (SD) from three independent experiments. To compare statistically significant differences, one-way ANOVA analysis and Dunnett’s post hoc test were utilized. The values of *p* < 0.05 were considered statistically significant using Prism 8 (GraphPad Software, San Diego, CA, USA).

## 3. Results

### 3.1. Effect of Treatment with HBX-5 Components on Viability of RWPE-1 and WPMY-1 Cells

**Figure 1:**
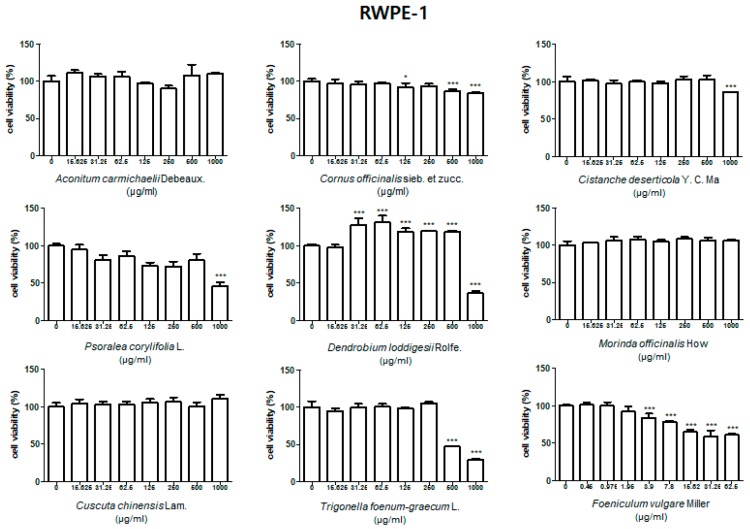
Effect nine herbs of HBX-5 on the cell viability in RWPE-1 cells. RWPE-1 cells were treated with 0.25–1000 μg/mL of HBX-5 components for 24 h. Values of cell viability were analyzed by ANOVA and Dunnett’s post hoc test for significances between each experimental group and resented as mean ± S.D. of three independent experiments (* *p* < 0.05, *** *p* < 0.001 versus untreated control group).

**Figure 2:**
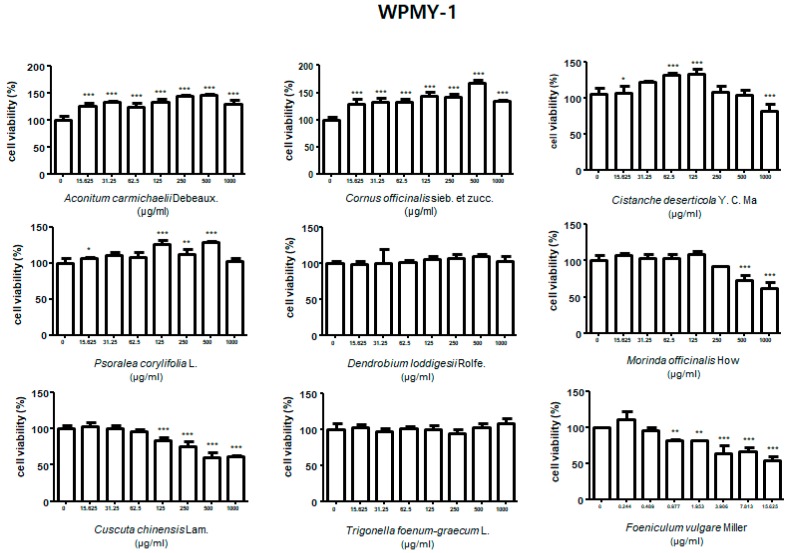
Effect of nine herbs of HBX-5 on the cell viability in WPMY-1 cells. WPMY-1 cells were treated with 0.25–1000 μg/mL of HBX-5 components for 24 h. Values of cell viability were analyzed by ANOVA and Dunnett’s post hoc test for significances between each experimental group and resented as mean ± S.D. of three independent experiments (* *p* < 0.05, ** *p* < 0.01, *** *p* < 0.001 versus untreated control group).

To evaluate the effect of HBX-5 components on the viability of prostate cells, we performed MTT assay in the normal prostate epithelial cell line RWPE-1, and the stromal cell line, WPMY-1, both of which were used to establish the in vitro BPH model. As shown in Figure 1 and Figure 2, treatment with HBX-5 components except *Foeniculum vulgare* Miller showed no toxicity in the dose range of 15.6–250 μg/mL in RWPE-1 and WPMY-1 cells. Since treatment with *Foeniculum vulgare* Miller (15.6–250 μg/mL) exhibited significant cell toxicity, we lowered its dose and investigated its effect on cell viability. Treatment with *Foeniculum vulgare* Miller (0.45–7.8 μg/mL) had no significant cell toxicity in RWPE-1 cells, and a dose range of 0.244–7.813 μg/mL showed no toxicity in WPMY-1 cells.

### 3.2. Effect of Treatment with HBX-5 Components on AR Expression in RWPE-1 and WPMY-1 Cells

**Figure 3:**
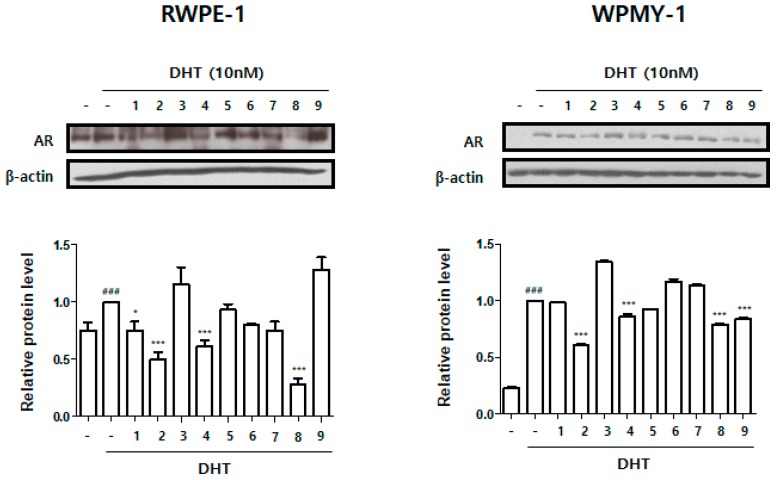
Effect of nine herbs of HBX-5 on the AR expression in human normal prostate cell. The normal human prostatic epithelial cell line, RWPE-1 and normal human prostatic stromal cell line, WPMY-1 were treated with 10 nM DHT for 72h and 24hr respectively, with or without each herb of HBX-6. 1, *Aconitum carmichaelii* 250 μg/mL; 2, *Cornus officinalis* Sieb. et Zucc. 250 μg/mL; 3, *Cistanche deserticola* Y. C. Ma 250 μg/mL; 4, *Psoralea corylifolia* L. 250 μg/mL; 5, *Dendrobium loddigesii* Rolfe. 250 μg/mL; 6, *Morinda officinalis* How 250 μg/mL; 7, *Cuscuta chinensis* Lam. 250 μg/mL; 8, *Trigonella foenum-graecum* L. 250 μg/mL; 9, *Foeniculum vulgare* Miller *2* μg/mL; β-actin was used as a loading control. The protein band densities were obtained by densitometric analysis. Density values were presented as mean ± S.D. (### *p* < 0.001 versus untreated control group; * *p* < 0.05, ** *p* < 0.01, *** *p* < 0.001 versus DHT-stimulated group).

Based on cell viability analysis by MTT assay, we investigated the effects of respective components of HBX-5 on AR expression in DHT-stimulated RWPE-1 and WPMY-1 cells. As shown in Figure 3, the expression of AR was significantly upregulated in response to DHT versus the untreated control group. In RWPE-1 cells, treatment with *Aconitum carmichaelii* Debeaux, *Cornus officinalis* sieb. et Zucc., *Psoralea corylifolia* L., and *Trigonella foenumgraecum* L. showed significant reduction in AR protein expression levels. Treatment with *Cornus officinalis* sieb. et Zucc., *Psoralea corylifolia* L., *Trigonella foenumgraecum* L., and *Foeniculum vulgare* Miller downregulated the expression of AR in WPMY-1 cells. Based on these results, we selected *Cornus officinalis* sieb. et Zucc., and *Psoralea corylifolia* L. for reconstitution of the new experimental formula, HBX-6.

### 3.3. Chemical Profiling Analysis of the Newly Combined Herbal Formula, HBX-6

**Figure 4:**
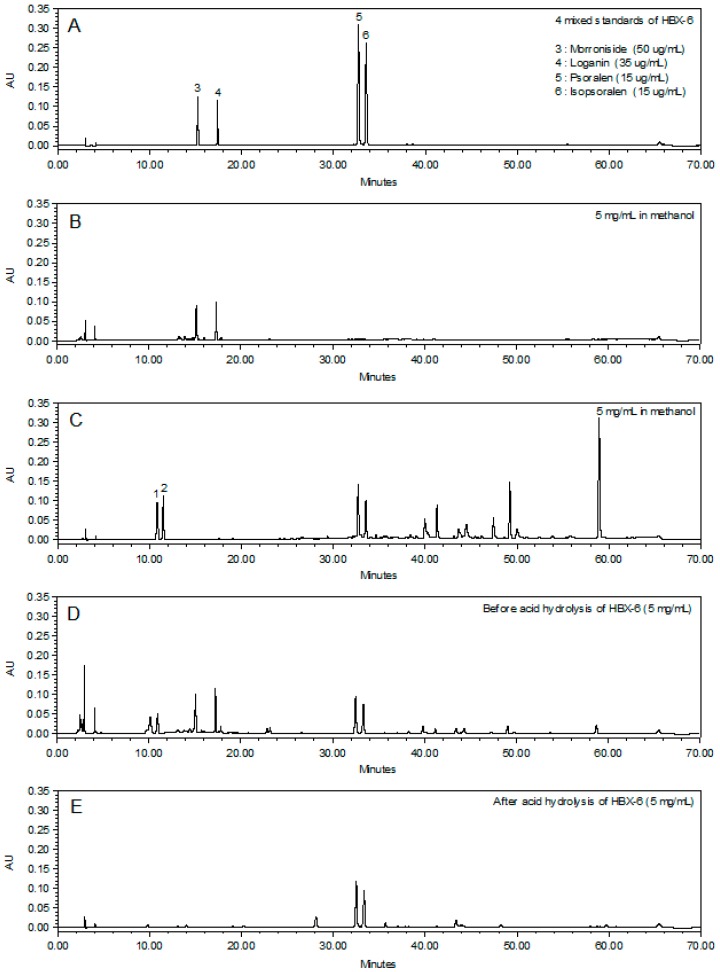
Representative HPLC chromatograms of (**A**) mixed standards, (**B**) *Cornus officinalis* Sieb. et Zucc., (**C**) *Psoralea corylifolia* L., (**D**) HBX-6 before acid hydrolysis and (**E**) HBX-6 after acid hydrolysis. (1) psoralenoside; (2) isopsoralenoside; (3) morroniside (4) loganin (5) psoralen; (6) isopsoralen. The six analytes have very broad range of polarity and various mobile phase compositions were tested. The mixture of 10 mM ammonium acetate (*w*/*v*) aqueous acetic acid and acetonitrile was finally chosen as the preferred mobile phase the reason why it produced the desired separation and acceptable tailing factors within the 70 min run time. Ground on the ultraviolet absorption characteristics of major compounds, the chromatograms were recorded at a wavelength of 290 nm. The separation was optimized when the column temperature was kept at 35 °C.

Representative spectrometry data of the mixed standards and the herbal extracts are shown in Figure 4. The persistently high contents of psoralen and isopsoralen were represented by acid hydrolysis as psoralenoside and isopsoralenoside, respectively. The six standard compounds were prepared to establish the calibration curves constructed by plotting the mean peak area versus the standard concentrations. As shown in Table 1, all standard curves show a suitable linear regression (r ≥ 0.9980) over the tested range, and then the limits of detection (LOD) and limits of quantification (LOQ) were analyzed for each compound. The identification of investigated compounds was carried out by comparison of their retention time and UV spectra with injected standards in the same conditions. The contents of four investigated compounds of *Cornus officinalis* sieb. et Zucc, *Psoralea corylifolia* L. and HBX-6 were summarized in Table 2.

### 3.4. Effect of HBX-6 Treatment on Prostate Weight in BPH-Induced Mouse Model

**Figure 5:**
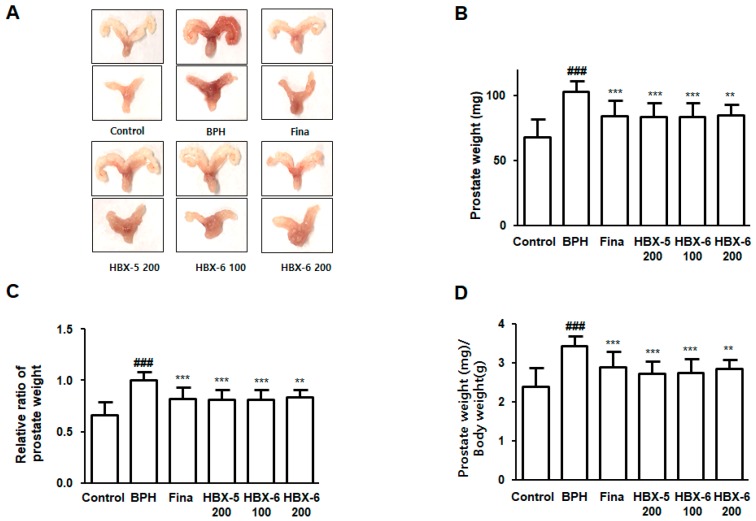
Effect of HBX-6 on prostate weight and prostate index in BPH-induced mice models. (**A**) A photographic assessment of prostatic enlargement from each experimental group is revealed. Change in (**B**) total prostate weight of the mice, (**C**) relative prostate weight ratio and (**D**) PW/BW ratio was assessed for the control, BPH, Fina, HBX-5 200 mg/kg, HBX-6 100 mg/kg and 200 mg/kg groups. Values were presented as mean ± SD (*n* = 10); ### *p* < 0.001 versus control group; * *p* < 0.05, ** *p* < 0.01, *** *p* < 0.001 versus the BPH group; ANOVA and Dunnett’s post hoc test were used for significances between each experimental group.

As shown in Figure 5, we investigated whether newly combined and simplified herbal formula, HBX-6 inhibited prostate enlargement in mice with BPH. Initially, we evaluated the macroscopic parameters of BPH and we observed that the BPH group showed significant prostatic enlargement and congestion compared with all other groups (Figure 5A). After sacrificing the mice, we resected the prostate from surrounding tissues and measured the weight and calculated the relative prostate weight (PW) ratio and PW/BW index. The results showed that BPH group showed a significant increase in prostate weight, relative prostate weight ratio, and PW/BW index. In comparison to the induced BPH group, mice treated with finasteride, HBX-5 (200 mg/kg), HBX-6 (100 mg/kg), and HBX-6 (200 mg/kg) significantly suppressed the overgrowth of prostate by 15%, 17%, 21%, and 20%, respectively (Figure 5B–D).

### 3.5. Effect of HBX-6 on Histological Changes in Prostate of BPH-Induced Mice

**Figure 6:**
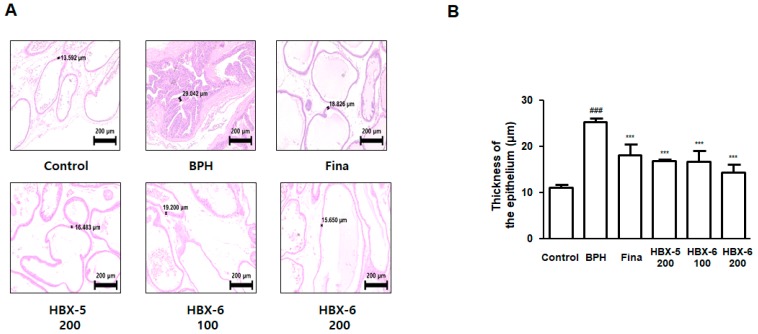
Effect of HBX-5 on the prostatic cell proliferation. (**A**) Prostatic tissue section from mice with BPH was stained with H&E; original magnification 100×. (**B**) Thickness of prostatic epithelial tissue. TETP was measured and represented as the mean ± SD (*n* = 5 for all experimental group). TETP was quantified at three section per each slide; ### *p* < 0.001 versus control group; *** *p* < 0.001 versus BPH group. ANOVA and Dunnett’s post hoc test were used for significances between each experimental group.

In order to investigate whether the inhibition of prostatic enlargement correlated with cellular hypertrophy, we performed histological analysis and quantified the thickness of prostatic epithelial tissue (TETP). As seen in Figure 6, mice with BPH showed a typical pattern of hyperplasia and hypertrophy, proliferation of the epithelial cells bulging into the luminal area, and the appearance of multilayered epithelial areas. However, mice treated with finasteride, HBX-5 (200 mg/kg), HBX-6 (100 mg/kg), and HBX-6 (200 mg/kg) showed amelioration of testosterone-treated prostate structures, although certain minor structural features of induced BPH remained. The TETP values in mice with BPH showed a 2.27-fold increase over mice from control group. Notably, the TETP values from finasteride, HBX-5 (200 mg/kg), HBX-6 (100 mg/kg), and HBX-6 (200 mg/kg) treated mice were significantly lower than those in BPH-induced mice by 28.58%, 33.46%, 34.15% and 43.42%, respectively.

### 3.6. Effect of HBX-6 on Prostate Cell Proliferation in BPH-Induced Mice

**Figure 7:**
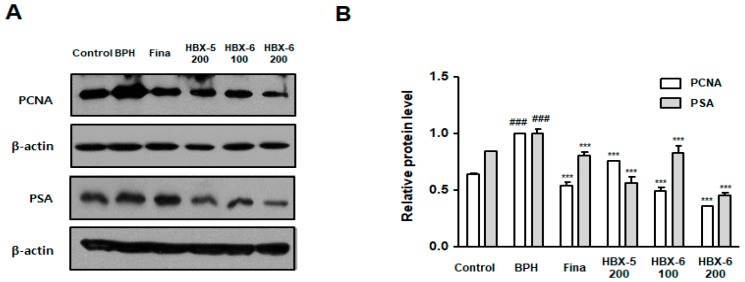
Effect of HBX-6 on proliferation-relative protein expression in prostate tissues of mice with BPH. The levels of PCNA and PSA were analyzed by a Western blot analysis. Values were presented as mean ± SD (*n* = 10); ### *p* < 0.001 versus control group; *** *p* < 0.001 versus BPH group. ANOVA and Dunnett’s post hoc test were used for significances between each experimental group.

PCNA is expressed in proliferating cells throughout the S-phase of the cell cycle and also shows increased expression in BPH human tissues [12]. Previous studies have also reported that PSA is a representative biomarker for prostate cancer but can also be used for the diagnosis of BPH, providing crucial information about cell proliferation and overgrowth [13]. In corroboration with previous reports, we showed that mice with BPH showed enhanced expression of PCNA and PSA. However, finasteride, HBX-5 (200 mg/kg), HBX-6 (100 mg/kg) and HBX-6 (200 mg/kg)-treated mice groups showed decreased expression of prostate cell proliferation-related proteins. HBX-5 and HBX-6 particularly, rescued the protein expression of PSA to the levels similar to control group (Figure 7).

### 3.7. Effect of HBX-6 on Cell Cycle-Related Proteins in BPH-Induced Mice

**Figure 8:**
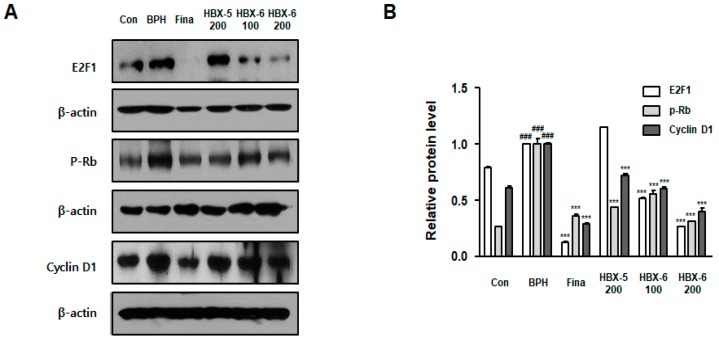
Effect of HBX-6 on cell cycle related-protein expression in prostate tissues of mice with BPH. The levels of E2F1, p-Rb and Cyclin D1 were analyzed by a Western blot analysis. Values were presented as mean ± SD (*n* = 10); ### *p* < 0.001 versus control group; *** *p* < 0.001 versus BPH group. ANOVA and Dunnett’s post hoc test were used for significances between each experimental group.

E2F1 and cyclin D1 are important players in the Cdk-Rb signaling pathway and are mainly involved in the transition of the cell cycle from G1 to S [14]. As shown in Figure 8, the BPH-induced mice group showed higher expression of E2F1, Rb and cyclin D1 as compared to the control mice, and the increased expression of these cell cycle-associated proteins was rescued by finasteride, HBX-5 (200 mg/kg), HBX-6 (100 mg/kg), or HBX-6 (200 mg/kg) treatments. We observed that the inhibitory effect of HBX-6 was markedly better than that of HBX-5. These results suggested that HBX-6 plays a critical role in the alleviation of BPH by the inhibition of cell cycle-associated proteins and related cellular signaling (Figure 8).

## 4. Discussion

As they age, men commonly develop BPH. About 50% of men in their 50s, and 80% of men in their 80s show symptoms of BPH. Usually, BPH is diagnosed based on clinical characteristics of benign prostatic enlargement and/or LUTS, and erectile dysfunction [7]. Prostate enlargement usually accompanies pathological symptoms and complications; therefore, prostate volume has been focused on in studies to understand the etiology, clinical pathology, and treatment of BPH [15]. In relation to this, an investigation of the effective medical treatment for BPH has been carried out over the last 25 years. Although invasive surgical intervention is generally considered as the gold standard of treatment for men with prostatic problems, shifts from transurethral resection of prostate to medication therapy management have been observed over time [16].

Therapy with the 5α reductase inhibitor, finasteride is a commonly used option for both, LUTS and BPH. A number of studies have shown that 5α reductase inhibitors have better safety and efficacy than α1-adrenergic receptors [17]. Despite their long clinical success, 5α reductase inhibitors show problems in clinical use. Treatment with 5α reductase inhibitors may lead to increased cardiovascular risk, breast cancer, and progression into high-grade prostate cancer. Especially, the recent data based on post marketing surveillance with 5α reductase inhibitors showed persisting adverse effects beyond drug seponation [18].

Herbal agents have been the subject of many studies in an effort to overcome the unintended consequences of chemical synthetic drugs in various diseases. As BPH is a chronic disease, the use of herbal medicine with efficacy on chronic conditions is deemed appropriate. Based on empirical experience, we developed a new formula composed of nine medicinal herbs and subsequently, we assessed its safety and efficacy on BPH-induced mice and cell lines. However, due to the drawbacks associated with the manufacturing process, we simplified and visualized the contents of HBX-5.

In this study, DHT-stimulated normal epithelial prostate RWPE-1 cells and normal stromal prostate WPMY-1 cells were used to assess the inhibitory effect of nine individual herbs on AR expression. Physiologically, androgens and AR are necessary to develop and preserve the male phenotype and male genitals. During the progression of BPH, AR is required for the proliferation of epithelial and stromal cells, thereby leading to prostate enlargement with obstructive uropathy [19]. Based on the evaluation of AR expression in prostate cells and empirical reports from oriental medicine, we selected *Cornus officinalis* and *Psoralea corylifolia*, and then reconstituted, standardized a new experimental formula, HBX-6.

The fruit of *Cornus officinalis sieb. et zucc.* is one of the primary herbs used in traditional Chinese medicine (TCM) [20]. Previous study reported that *Cornus officinalis* has the therapeutic effect on sexual dysfunction, and cornuside, an isolated compound from *Cornus officinalis*, has vascular relaxation activity, suggesting an enhancement of sexual function [21]. *Psoralea corylifolia* L., has been traditionally used in treatment of impotence, oliguria premature ejaculation in Korean medicine [22]. A recent study has detailed the therapeutic effect of *Psoralea corylifolia* L. on spermatogenesis in rat testes [23].

Although an in vivo BPH model is necessary to evaluate the efficacy of medication, spontaneous BPH with increasing age is unusual in animals except humans. A testosterone-induced BPH mouse model is therefore a good substitute model that mimics morphological changes in human prostate hyperplasia [24,25].

In this study, BPH-induced mice notably manifested the pathological alterations in the prostate and surrounding tissues. Prostatic enlargement and congestion in the BPH-induced mice showed a clear distinction as compared to the mice in the control group and treatment groups (Figure 5). Abnormal cellular proliferation in the prostate tissue has been considered one of the most important events in the progression of BPH [26]. Using histological analysis, we observed an increased thickness of the epithelium due to pathologically activated epithelial cell proliferation in the prostate tissue of BPH-induced mice. In Hematoxylin and eosin (H & E) stained prostate tissue sections, the BPH-induced mice displayed reduced glandular luminal area and typical hypertrophic patterns as compared to the control group, whereas treatment with finasteride, HBX-6 (200 mg/kg), HBX-5 (100 mg/kg), and HBX-5 (200 mg/kg) reduced the severity of histological features of BPH (Figure 6).

Patients with higher PSA levels suffer from progressive BPH, and PSA levels accurately reflect the prostate size and can be used as a reliable parameter with potential foreseeable efficacy as a marker in clinical treatment for BPH [27]. Along with PSA, PCNA, expressed in proliferating cell nuclei, is implicated in the progression of BPH. PCNA is an adminicular factor that is intertwined with cyclin D1-CDK complex [12]. In the present study, we noted that HBX-6 (100 mg/kg) and HBX-6 (200 mg/kg) reduced the expression of PCNA and PSA in testosterone-induced BPH mice (Figure 7).

There has been an increasing interest in exploring the underlying causes and mechanisms that are intricately related to the development and progression of BPH. The transcription factor E2F family of proteins is an important factor in the regulation of cell proliferation and apoptosis. The E2F family consists of eight members, which are classified as activators (E2F1, E2F2, E2F3) or repressors (E2F4, E2F5, E2F6, E2F7, E2F8), based on their ability to either activate or repress transcription [28,29]. E2F family mainly regulates the G1/S transition during cell cycle by regulating the transcription of cell cycle-related genes, such as cyclin A, cyclin E, and PCNA [30]. During G1/S transition, cyclin D-cdk4/6 complex phosphorylates Rb and allows its disassembly from E2F, which further activates the transcription of S-phase genes [14]. Among E2F family members, overexpression of E2F activators induces entry into S-phase, activates DNA synthesis, and also facilitates the transcriptional activation of E2F-target genes. E2F activators also can negate growth arrest signals initiated by the Cdk inhibitors [30,31]. Recently, Liang et al. reported that the overexpression of E2F1 promotes cell invasion and migration in prostate cancer cells [32].

Meanwhile, according to OECD guidelines for the testing of chemicals Section 4 health effects test, we performed a repeated dose 90-day oral toxicity study in rats. All animals were given HBX-6 daily at the dose of 1000, 2000 and 4000 mg/kg for 90 days and survived to the scheduled termination. Dose of 4000 mg/kg was decided as no observed effect level (NOEL) in male and female rats. Evidences from this study back up the possibility of HBX-6 as natural remedies for BPH.

An epidemiological study suggested that the development of BPH predisposes an individual to develop prostate cancer in his lifetime [33]. Similarly, previous studies have reported that the risk of prostate cancer is especially high in Asian males with BPH [34]. Although there are many different hypotheses about the relationship between prostate cancer and BPH, these two prostate diseases can be associated with each other by cellular and molecular mechanisms. Among them, an imbalance of cellular proliferation and apoptosis, and the associated molecular pathways have received attention in the management of prostate diseases. In this study, we observed that BPH-induced mice show an upregulation of E2F1, Rb, and cyclin D1. These results are consistent with previously reported evidence suggesting a connection between cyclin D1 and E2F1 expression in BPH. Interestingly, we showed that HBX-6 treatment significantly reduced the expression levels of E2F1, Rb, and cyclin D1 in comparison with BPH-induced mice (Figure 8).

## 5. Conclusions

The most significant finding of this study is that the simplified herbal agent HBX-6 has a strong therapeutic potential in the suppression of BPH via the inhibition of the E2F1 pathway in a testosterone-induced BPH mouse model. In addition, these results showed that the inhibitory effect of HBX-6 on prostatic cell proliferation is superior to that of positive control agents, finasteride, and HBX-5 (200 mg/kg). Taken together, we suggest the possibility of using HBX-6 as a therapeutic agent for BPH treatment.

## Figures and Tables

**Table 1:** Regression data, LODs and LOQs for the 4 analytes of the assay.

Analyte	Regression Equation	r2	Linear Ranage (μg/mL)	LOD ^a^ (μg/mL)	LOQ ^b^ (μg/mL)
Morroniside	y = 17572x − 9299.20	0.9981	25.00–125.00	2.75	0.91
Loganin	y = 16559x + 9546.56	0.9994	20.00–80.00	0.22	0.65
Psoralen	y = 85821x + 21320.47	0.9997	10.00–30.00	0.50	1.51
Isopsoralen (Angelicin)	y = 77196x − 61110.20	0.9986	10.00–30.00	0.30	0.10

^a^ LOD refers to the limits of detection. ^b^ LOQ refers to the limits of quantification.

**Table 2:** Contents of four bioactive ingredients in *C. officinalis*, *P. corylifolia* and HBX-6.

No.	Retension Time (min)	Analyte	*Cornus officinalis* Sieb. et Zucc.	*Psoralea corylifolia* L.	HBX-6
				Content (mg/g)	
(3)	15.197	Morroniside	7.490 ± 0.143	-	11.511 ± 0.015
(4)	17.368	Loganin	4.821 ± 0.082	-	8.001 ± 0.013
(5)	32.761	Psoralen	-	3.072 ± 0.004	3.457 ± 0.003 ^a^
(6)	33.613	Isopsoralen (Angelicin)	-	2.473 ± 0.002	3.110 ± 0.008 ^a^

^a^ after acid hydrolysis of HBX-6. Data is represented as the mean ± SD (*n* = 3).

## Abbreviations

BPH	Benign prostatic hyperplasia
DHT	dihydrotestosterone
LUTS	lower urinary tract symptoms
IHC	immunohistochemistry
H & E	hematoxylin and eosin
